# Methylation status of *COX-2* in blood leukocyte DNA and risk of gastric cancer in a high-risk Chinese population

**DOI:** 10.1186/s12885-015-1962-x

**Published:** 2015-12-16

**Authors:** Hui-juan Su, Yang Zhang, Lian Zhang, Jun-ling Ma, Ji-You Li, Kai-feng Pan, Wei-cheng You

**Affiliations:** 1 Key Laboratory of Carcinogenesis and Translation Research (Ministry of Education/Beijing), Department of Cancer Epidemiology, Peking University Cancer Hospital & Institute, Beijing, P.R. China; 2 Department of Pathology, Peking University Cancer Hospital & Institute, Beijing, P.R. China

**Keywords:** DNA methylation, Blood leukocyte, *COX-2*, Gastric cancer

## Abstract

**Background:**

Methylation is a common epigenetic modification which may play a crucial role in cancer development. To investigate the association between methylation of *COX-2* in blood leukocyte DNA and risk of gastric cancer (GC), a nested case–control study was conducted in Linqu County, Shandong Province, a high risk area of GC in China.

**Methods:**

Association between blood leukocyte DNA methylation of *COX-2* and risk of GC was investigated in 133 GCs and 285 superficial gastritis (SG)/ chronic atrophic gastritis (CAG). The temporal trend of *COX-2* methylation level during GC development was further explored in 74 pre-GC and 95 post-GC samples (including 31 cases with both pre- and post-GC samples). In addition, the association of DNA methylation and risk of progression to GC was evaluated in 74 pre-GC samples and their relevant intestinal metaplasia (IM)/dysplasia (DYS) controls. Methylation level was determined by quantitative methylation-specific PCR (QMSP). Odds ratios (ORs) and 95 % confidence intervals (CIs) were calculated by unconditional logistic regression analysis.

**Results:**

The medians of *COX-2* methylation levels were 2.3 % and 2.2 % in GC cases and controls, respectively. No significant association was found between *COX-2* methylation and risk of GC (OR, 1.15; 95 % CI: 0.70-1.88). However, the temporal trend analysis showed that *COX-2* methylation levels were elevated at 1–4 years ahead of clinical GC diagnosis compared with the year of GC diagnosis (3.0 % vs. 2.2 %, *p* = 0.01). Further validation in 31 GCs with both pre- and post-GC samples indicated that *COX-2* methylation levels were significantly decreased at the year of GC diagnosis compared with pre-GC samples (1.5 % *vs.* 2.5 %, *p* = 0.02). No significant association between *COX-2* methylation and risk of progression to GC was found in subjects with IM (OR, 0.50; 95 % CI: 0.18–1.42) or DYS (OR, 0.70; 95 % CI: 0.23–2.18). Additionally, we found that elder people had increased risk of *COX-2* hypermethylation (OR, 1.55; 95 % CI: 1.02–2.36) and subjects who ever infected with *H. pylori* had decreased risk of *COX-2* hypermethylation (OR, 0.54; 95 % CI: 0.34–0.88).

**Conclusions:**

*COX-2* methylation exists in blood leukocyte DNA but at a low level. *COX-2* methylation levels in blood leukocyte DNA may change during GC development.

## Background

Gastric cancer (GC) is the second leading cause of cancer death worldwide [1]. Evidences accumulatively revealed that GC was a consequence of multistage progression of gastric lesions with complex molecular alterations, including DNA methylation [2–4].

Several tumor-related genes, such as *CDH1*, *p16*, *APC*, *COX-2*, *RUNX3*, and *hMLH1*, were detected aberrant methylation in GC [5–8]. However, most of these studies were focused on tissue samples, and few data on the alteration of blood leukocyte DNA methylation was reported. Unlike tissue DNA, blood leukocyte DNA can be obtained non-invasively and inexpensively, thus, aberrant methylation of blood leukocyte DNA may serve as a potential biomarker for GC diagnosis.

Cyclooxygenase 2 (COX-2) is an inducible enzyme, and particularly overexpressed during inflammation of tissue [9]. Animal models showed that COX-2 played important roles in cell adhesion, apoptosis, and angiogenesis [10]. Recently, COX-2 was found to be up-regulated in various carcinomas and play a central role in tumorigenesis [11–13]. Our previous study demonstrated that overexpression of COX-2 was associated with *Helicobacter pylori* (*H. pylori*) infection and increased the risk of precancerous gastric lesions [14]. Studies *in vitro* and in tumor tissue suggested that promoter methylation status of *COX-2* may regulate mRNA and protein expression [8, 15–17]. However, little is known about *COX-2* promoter methylation status in blood leukocyte DNA.

In this study, we were particularly interested in the association between *COX-2* methylation in blood leukocyte DNA and risk of GC. We compared the *COX-2* methylation levels in GC cases with superficial gastritis (SG) or mild chronic atrophic gastritis (CAG) controls. In addition, blood samples collected before or/and after GC clinical diagnosis from two long-term cohorts provided us a unique opportunity to evaluate the dynamic changes of *COX-2* methylation levels during progression of gastric lesions and GC development.

## Methods

### Study population

In 1989 and 2002, two cohort studies were launched in Linqu County, involving 3433 and 2638 subjects [18, 19], and 186 GCs were identified until 2009. Endoscopic screening was performed at baseline of each cohort and followed a repeated endoscopic examination using the same procedures in 1999, 2003 and 2009, respectively. For each subject, the biopsy specimens were taken from 5–7 standard sites of the stomach, and given its corresponding histopathologic diagnosis by three senior pathologists independently from Peking University Cancer Hospital according to the Updated Sydney System [20] and Padova International Classification [21]. Each biopsy was classified according to the presence or absence of SG, mild/severe CAG, intestinal metaplasia (IM), dysplasia (DYS) or GC, and given a diagnosis based on the most severe histology. Each subject was assigned a “global” diagnosis based on the most severe diagnosis among any of the biopsies.

**Fig. 1:**
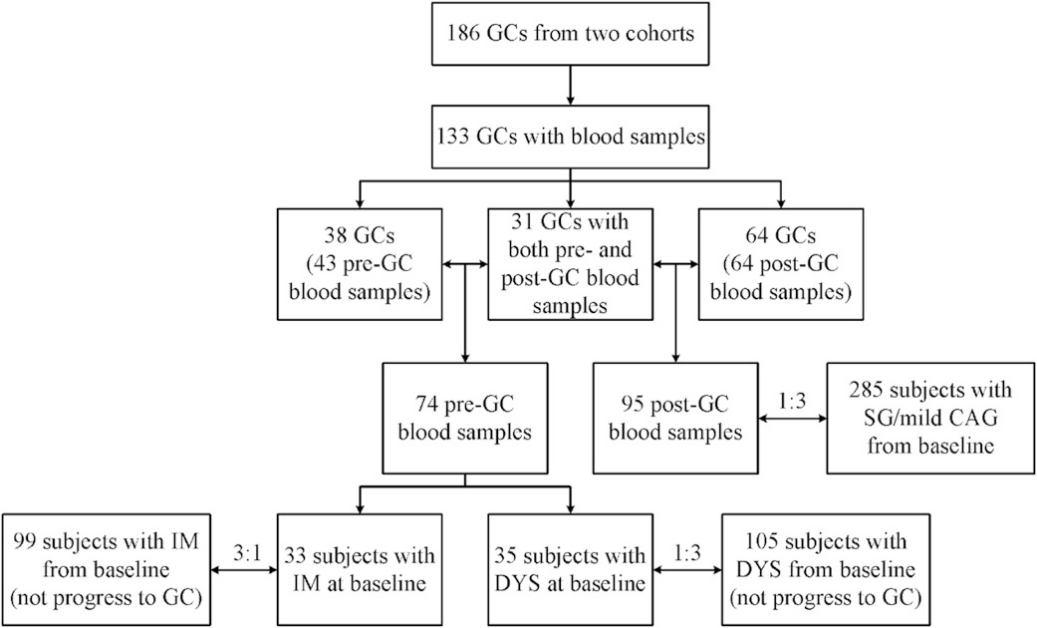
Structure of sample selection. All subjects were selected from our two cohort studies, including 133 GC cases, 285 SG/mild CAG, 99 IM and 105 DYSs

For the current study, a nested case–control design was used based on the two cohorts enrolling 133 GC cases with at least one blood sample from follow-up period. According to the time of diagnosis, blood leukocyte samples collected from GC cases were defined into pre-GC (before GC diagnosis ranging from 1 to 10 years) and post-GC (the year of GC diagnosis or up to 10 years after). Among them, 74 pre-GC blood samples from 69 GC cases (5 cases with two pre-GC samples with different time interval) and 95 post-GC samples were collected. Additionally, 31 cases had both pre-GC and post-GC samples were also selected as self-control to measure the methylation levels in the two time intervals (Fig. 1).

To test *COX-2* methylation level and risk of GC, 285 subjects with SG or mild CAG were selected as controls for 95 post-GC cases at random with a ratio of 1:3 and frequency-matched in age category (<60 and ≥60 years) and gender. We further selected 99 subjects with IM and 105 with DYS who did not progress to GC during the follow-up period randomly from baseline as controls, because the corresponding gastric lesions for the pre-GC diagnosis were mainly IM (*n* = 33) and DYS (*n* = 35) (Fig. 1).

All of the blood samples were collected before the endoscopic examination. Information on gender, date of birth, cigarette smoking and alcohol drinking were obtained from the questionnaires at the baseline of the two cohorts, respectively. Age was determined according to the year when blood sample was collected. Because a number of repeated endoscopic examinations were performed, more than one blood samples from the same subject were collected. Consequently, different ages were calculated corresponding to the date of sample collection in the data analysis. This study was approved by the Institutional Review Board of Peking University School of Oncology and all subjects gave written informed consent.

### DNA preparation and bisulfite modification

Peripheral blood samples were collected in K_2_EDTA tubes (BD Vacutainer®) and centrifuged at 3000 rpm for 10 min for separation from plasma. The leukocyte fraction was washed by Tris-EDTA for 3 times and high molecular weight genomic DNA was isolated by standard proteinase K digestion and phenol-chloroform extraction. Bisulfite treatment was reported previously [22]. Briefly, 1–10 μg genomic DNA was modified with sodium bisulfite for 16 h at 50 °C to completely convert the unmethylated cytosines to uridines. Bisulfite treated DNA was then purified with a genomic DNA purification kit (Promega, Madison, WI) and stored at −20 °C until use.

### *COX-2* Methylation analysis

Fluorescence-based, real-time quantitative methylation-specific PCR (QMSP) was carried out for *COX-2* using a 7500 fast Real-time PCR system (Applied Biosystems, Foster City, CA, USA) with the primers and probe as described previously [23]. The PCR was conducted in a 20-μl mixture, containing 100 ng of bisulfate modified DNA, 200nM of each primer and probe, and 10 μl 2X-MaximaTM Probe/Rox qPCR Master Mix (Fermentas Burlington, Ontario, Canada) at the following conditions: 95 °C for 10 min, followed by 40 cycles of 95 °C for 15 s and 60 °C for 1 min. The efficiency of PCR amplification was confirmed to be nearly 100 %, and beta actin (*ACTB*) was used as a reference set to normalize for input DNA.

The methylation level of *COX-2* was expressed as percentage, calculated by dividing the *COX-2*/*ACTB* ratio of a sample by the *COX-2*/*ACTB* ratio of HL60 (a human promyelocytic leukemia cell line which was confirmed to be 100 % methylated in the CpGs in *COX-2* primers and probe). The analysis was performed blind by one technician, and various lesion groups were randomly mixed for bisulfite treatment and real-time PCR. Each primer pair was run in a separate well and at least 2 parallels were required at each sample. Parallels were removed when the CT values differed more than 0.06, and the same sample was repeated. A total unmethylated cell line MKN45 was used as negative control to qualify the PCR reaction as well as DNA preparation and bisulfite modification procedure.

### *H. pylori* antibody assay

*H. pylori* antibody assays were used for determination of *H. pylori* infection with the serum separated from blood samples collected. Details of serologic assay were described previously [24]. Briefly, serum levels of anti-*H. pylori* IgG were measured separately in duplicate with enzyme-linked immunosorbent assay (ELISA) procedures. An individual was determined to be positive for *H. pylori* infection if the mean optical density of IgG ≥ 1.0. Quality-control samples were assayed at Vanderbilt University, Nashville, Tennessee.

### Statistical analysis

Pearson’s *χ*^*2*^ test was used to examine the differences in distribution of age group, gender, smoking, drinking and *H. pylori* infection status between SG/CAG and post-GC groups. Mann–Whitney/Wilcoxon test was used to compare the *COX-2* methylation levels between SG/CAG and post-GC groups.

Odds ratios (ORs) and 95 % confidence intervals (CIs) were used to assess the associations between *COX-2* methylation and the risk of GC and progression of gastric lesions, the potential risk factors, and the differences methylation levels between pre-GC and post-GC groups by unconditional logistic regression, adjusting for age, gender, smoking, drinking, and *H. pylori* infection status. *P*_trend_ was applied by unconditional logistic regression to analyze the temporal trend of *COX-2* methylation levels. To compare the methylation status in 31 GC cases with both pre- and post-diagnosis blood samples, conditional logistic regression was applied with age adjusted.

All analyses were performed using the Statistical Analysis System software (version 9.0; SAS Institute, Cary, NC). *P* value of <0.05 was considered significant and all statistical tests were two sided.

## Results

The frequency distributions of age, gender, cigarette smoking, alcohol consumption and *H. pylori* status of 95 post-GCs and 285 controls were presented in Table 1. The frequency of *H. pylori* infection was significantly higher in GC than control group (88.4 % *vs.* 61.4 %, *p* < 0.001). The other factors showed no statistical difference in the two groups.

**Table 1:** Selected characteristics of the individuals

Variables	Post-GC	SG/mild CAG	*P* ^*a*^
	*n* = 95	*n* =285	
Age (%)			1.00
< 60	40(42.1)	120(42.1)	
≥ 60	55(57.9)	165(57.9)	
Gender (%)			1.00
Female	26(27.4)	78(27.4)	
Male	69(72.6)	207(72.6)	
Smoking (%)			0.99
Ever smoke	57(60.0)	173(60.7)	
Never smoke	37(38.9)	112(39.3)	
Missing	1(1.1)		
Drinking (%)			0.93
Ever drink	48(50.5)	154(54.0)	
Never drink	40(42.1)	131(46.0)	
Missing	7(7.4)		
*H. pylori* infection (%)			<0.001
Ever infected	84(88.4)	175(61.4)	
Never infected	11(11.6)	110(38.6)	

^a^ χ^2^ test, *P* value for each covariate was estimated among participants without missing value in that variate

### Methylation levels in GCs and SG/CAG controls

We first compared the methylation levels of *COX-2* between GC cases and SG/mild CAG controls. The medians (interquartile range) of *COX-2* methylation levels were 2.3 % (1.2–3.9 %) in cases and 2.2 % (1.4–3.4 %) in controls (*p* = 0.94). To further evaluate the relationship between *COX-2* methylation and risk of GC, we set 2 % as a cut-off value according to the median level in control group. No significant association was found between *COX-2* methylation level and GC risk (OR, 1.15; 95 % CI: 0.70–1.88) after adjusting for age, gender, smoking, drinking and *H. pylori* infection.

### Temporal trends of methylation levels in GC development

By comparing pre-GC (*n* = 74) and post-GC (*n* = 95) samples (Table 2), we found that *COX-2* methylation levels were slightly lower in post-GC samples than pre-GC samples (2.3 % *vs.*2.5 %), although the p value showed no statistical significance (*p* = 0.32).

**Table 2:** The temporal trends of COX-2 methylation levels during GC development

	n	Methylation proportion	*P* ^a^
		Median % (interquartile range)	
Total pre-GC and post-GC samples			
Pre-GC	74	2.5(1.5–4.4)	
Post-GC	95	2.3(1.2–3.9)	
*P* ^b^		0.32	
Temporal trend			
5–10 years pre-GC	32	1.9(1.4–4.0)	0.53
1–4 years pre-GC	42	3.0(2.0–4.5)	0.01
GC diag. year	46	2.2(1.1–2.8)	Ref.
1–4 years post-GC	21	1.9(1.4–2.9)	0.80
5–10 years post-GC	28	2.8(1.8–4.9)	0.06
*P*_trend_^c^		0.32	

^a^Mann-Whitney Test/Wilcoxon Test

^b^ Unconditional logistic regression analysis, adjusted for age, gender, smoking, drinking and *H. pylori* infection status

^c^ Unconditional logistic regression analysis

The temporal trend of *COX-2* methylation levels during GC development was explored by dividing the pre- and post-GC samples into 5 groups (5–10 years pre-GC, 1–4 years pre-GC, GC diagnosis year, 1–4 years post-GC and 5–10 years post-GC) according to the time interval between sample collection and GC diagnosis. As shown in Table 2, the median methylation levels of *COX-2* in different groups were 1.9 % (1.4–4.0 %), 3.0 % (2.0–4.5 %), 2.2 % (1.1–2.8 %), 1.9 % (1.4–2.9 %) and 2.8 % (1.8–4.9 %), respectively. Taking the year of GC diagnosis as reference (2.2 %), *COX-2* methylation levels were significantly increased at 1–4 years ahead of clinical GC diagnosis (3.0 %, *p* = 0.01), and decreased at 1–4 years after GC diagnosis (1.9 %, *p* = 0.80). However, *COX-2* methylation was back to a higher level at 5–10 years after GC diagnosis (2.8 %, *p* = 0.06). Since *COX-2* methylation levels fluctuated before and after GC clinical diagnosis, we did not find a significance linear trend between groups (*p* = 0.32).

A similar trend of *COX-2* methylation levels was further validated in 31 GC cases (10 females and 21 males) with both pre-GC and post-GC samples (Table 3). We found that *COX-2* methylation levels were significantly decreased in post-GC compared with pre-GC samples (1.5 % *vs.* 2.5 %, *p* = 0.04). Because most of the 31 pairs of GC samples were collected at 1–4 years ahead of diagnosis (*n* = 22) and GC diagnosis year (*n* = 21), we compared two groups and found that *COX-2* methylation levels were significantly lower in GC diagnosis year samples than in 1–4 years pre-GC samples (1.5 % *vs.*2.5 %, *p* = 0.02).

**Table 3:** The methylation level in 31 pairs of GC cases

	Methylation proportion
	Median % (interquartile range)
Self-control study	
Pre-GC	2.5(1.4–4.4)
*n* = 31	
Post-GC	1.5(0.9–2.8)
*n* = 31	
*P* ^a^	0.04
Temporal trend	
1–4 years pre-GC	2.5(1.4–4.5)
*n* = 22	
GC diag. year	1.5(0.7–2.7)
*n* = 21	
*P* ^b^	0.02

^a^ Conditional logistic regression analysis, adjusted for age

^b^ Mann–Whitney Test/Wilcoxon Test

### Methylation levels in IM or DYS subjects with different outcomes

Because the corresponding gastric lesions for the pre-GC diagnosis were mainly IM and DYS, we were very interested to compare the methylation levels in subjects with IM or DYS progressed or not progressed to GC during the follow-up period. However, no significant differences were found between subjects with IM/DYS progressed or not to GC (OR, 0.50; 95 % CI: 0.18–1.42 for IM and OR, 0.70; 95 % CI: 0.23–2.18 for DYS) (Table 4).

**Table 4:** Association between COX-2 methylation and risk of progression to GC

	Hypermethylated^a^	Hypomethylated	OR(95 % CI)^b^	*P* ^b^
IM (Progress to GC)	18(54.6)	15(45.5)	0.50(0.18-1.42)	0.19
*n* = 33				
IM (Not progress to GC)	69(69.7)	30(30.3)		
*n* = 99				
DYS (Progress to GC)	24(68.6)	11(31.4)	0.70(0.23–2.18)	0.54
*n* = 35				
DYS (Not progress to GC)	70(66.7)	35(33.3)		
*n* = 105				

^a^ Cut-off value was set as 2 %, according to the median COX-2 methylation level of SG/CAG group

^b^ Unconditional logistic regression analysis, adjusted for age, gender, smoking, drinking and *H. pylori* infection status

### Relationships between methylation status and epidemiologic parameters

We also examined the association between *COX-2* methylation level and age or other risk factors. As shown in Table 5, for the total participants, *COX-2* methylation levels were significantly higher in older subjects (OR, 1.55; 95 % CI: 1.02–2.36), but lower in subject who ever infected with *H. pylori* (OR, 0.54; 95 % CI: 0.34–0.88). No statistically significant associations were observed between *COX-2* methylation level and gender, smoking, and drinking.

**Table 5:** Factors affecting blood leukocyte methylation of COX-2

Characteristics	Total (*n* = 380)		SG/mild CAG (*n* = 285)		Post-GC (*n* = 95)	
	n	OR (95 % CI)^a^	n	OR (95 % CI)^a^	n	OR (95 % CI)^a^
Age						
< 60	160	1.00	120	1.00	40	1.00
> =60	220	1.55(1.02–2.36)	165	1.54(0.94–2.51)	55	1.77(0.71–4.39)
Gender						
Female	104	1.00	78	1.00	26	1.00
Male	276	1.34(0.71–2.53)	207	1.57(0.74–3.31)	69	0.90(0.25–3.20)
Smoking						
Never	149	1.00	112	1.00	37	1.00
Ever	230	0.99(0.56–1.73)	173	0.89(0.47–1.69)	57	1.21(0.37–3.98)
Current	205	0.96(0.57–1.64)	155	0.84(0.46–1.54)	50	1.40(0.45–4.39)
Drinking						
Never	171	1.00	131	1.00	40	1.00
Ever	202	0.70(0.43–1.16)	154	0.61(0.34–1.09)	48	1.02(0.35–3.00)
Current	182	0.74(0.46–1.20)	138	0.64(0.37–1.11)	44	1.22(0.43–3.43)
*H. pylori* infection						
Never	121	1.00	110	1.00	11	1.00
Ever	259	0.54(0.34–0.88)	175	0.49(0.29–0.83)	84	1.18(0.28–4.98)
Current	210	0.71(0.47–1.09)	150	0.69(0.43–1.12)	60	0.77(0.31–1.93)

^a^ Unconditional logistic regression analysis, adjusted for other factors (age, gender, smoking, drinking or *H. pylori* infection status)

## Discussion

In the present study, based on our two cohort studies in a high-risk population of GC, we quantified *COX-2* methylation level in blood leukocyte DNA of various gastric lesions and investigated the relationship between methylation of *COX-2* in blood leukocyte DNA and risk of GC.

Until now, studies on the association between blood leukocyte DNA methylation and risk of GC are limited. Several studies suggested that global hypomethylation in blood leukocyte DNA may be related to GC risk [25, 26]. Recently, a study showed that whole blood *p16* methylation may serve as an important prognostic indicator of gastric adenocarcinoma [27]. A Japanese study showed that methylation level of *IGF2* in blood leukocyte DNA was lower in GC cases than healthy controls [28]. To our best knowledge, this is the first study to explore the relationship of *COX-2* methylation in blood leukocyte DNA and risk of GC.

Human *COX-2* gene is located in 1q25.2–25.3, consisting of 10 exons and 9 introns. In the 5′-flanking region, there is a CpG island containing several potential transcription factor binding sites, including two NF-κB sites, two AP-2 sites, three SP1 sites, one C/EBP motif, one Ets-1 site, and one CRE site [29]. SP1 and AP-2 were two human transcription factors, which play critical roles in regulating gene expression during embryonic early development [30–35]. We selected a 75 bp region containing 7 CpG sites in the downstream of the transcriptional starting codon from −296 to −222 with one SP1 binding site and one AP-2 binding site.

In this study, we found that *COX-2* methylation existed in blood leukocyte DNA, but at a low level. The median of *COX-2* methylation levels was only 2.2 % in SG/mild CAG group. A previous study reported that the frequency of *COX-2* hypermethylation was 88 % in primary prostate cancer tissues [36]. However, a German study showed that the frequency of *COX-2* hypermethylation was only 2.4 % in serum of prostate cancer [37]. Another study using microdissected foci collected from esophageal cancer patients showed that *COX-2* methylation was more common in subepithelial lymphocytes than in epithelial foci or non-lymphocytic stromal tissues [38]. These findings suggested that *COX-2* methylation might have tissue specificity.

In the present study, we did not found association between *COX-2* methylation in blood leukocyte DNA and risk of GC. However, the temporal trend analysis showed that *COX-2* methylation levels were elevated at 1–4 years ahead of clinical GC diagnosis. Further validation using 31 GC cases with both pre- and post-GC blood samples indicated that *COX-2* methylation levels were significantly increased before GC diagnosis, suggesting that subjects with higher *COX-2* methylation levels in blood leukocyte DNA may increase the GC risk. However, no significant association between *COX-2* methylation and risk of progression to GC was found in subjects with IM and DYS who progressed to GC in contrast to those remained with IM and DYS. It may speculate that *COX-2* methylation levels mainly increased 1–4 years but not 5–10 years prior to clinical diagnosis. For subjects with IM or DYS who progressed to GC, the blood samples were collected not only at 1–4 years (18 IM, 20 DYS), but also at 5–10 years (15 IM, 15 DYS). Due to the small sample size, we cannot conduct a stratified analysis. Further study with a large sample size is warranted to confirm our results. In addition, because *COX-2* methylation levels in blood leukocyte DNA were very low, more studies are needed to identify potential biomarkers for GC diagnosis.

The mechanism for blood leukocyte DNA methylation of *COX-2* and risk of GC is still unclear. Until now, no study focused on the mechanism of blood leukocyte DNA methylation and carcinogenesis process, and whether DNA methylation levels in blood leukocytes could represent those in tissues was still unclear. Studies showed that *COX-2* mRNA and protein expression were frequently up-regulated in human GC tissue and cell lines [39–41], and 5-aza-deoxycytidine treatment could increase both *COX-2* mRNA and protein expression *in vitro* [42–44]. Another study found that treatment of *COX-2*-methylated cells with 5-azacytidine had a modest effect on COX-2 expression, but when 5-azacytidine-treated cells were subsequently stimulated with *H. pylori*, there was a significant, 5–10-fold enhancement of both *COX-2* mRNA and protein expression [9]. These findings suggested that *COX-2* methylation may be involved in gastric carcinogenesis via regulation *COX-2* mRNA and protein expression. However, the biological significance of blood leukocyte DNA methylation of *COX-2* needs further studies.

Growing evidences demonstrated that age, environment and lifestyle factors may modify DNA methylation [45–47]. Studies on specific gene methylation showed that *CDH1*, *p53*, *RUNX3*, *p16* methylation levels were significant higher in older persons [27, 48]. Aging is associated with global hypomethylation of DNA and hypermethylation of specific genes [49–51]. In our study, we found higher *COX-2* methylation levels in blood leukocytes in older persons, consistent with the hypothesis and previous studies. *H. pylori* infection was a well-known factor which was associated with methylation of many tumor-related genes [5, 52]. A study suggested that loss of *COX-2* methylation might facilitate COX-2 expression, which associates with *H. pylori* infection [9]. In the current study, we found that *COX-2* methylation levels were lower in subjects who ever infected with *H. pylori*. We were also interested in association between differentiation types, metastasis and surgery status of GC and *COX-2* methylation levels. Based on our available data, we found that subjects with poor differentiation, metastasis and without surgery had low methylation levels compared with those with moderate/high differentiation, without metastasis and surgery. However, no significant differences were found (data not shown).

Our study has several strengths. Firstly, all subjects came from a high-risk area of GC, containing various pathological diagnosed samples. Secondly, our study had pre-GC diagnosis blood samples for the dynamic observation of *COX-2* methylation and also for the comparison of methylation levels between subjects progressed and non-progressed to GC. Instead of normal controls, we selected SG/mild CAG subjects as references, however, this “sub-normal” control could only lead to the dilution of disparity between comparison groups. In addition, because of the limited number of GC cases (*n* = 31) with both pre- and post-GC samples, unmatched samples were also analyzed for COX-2 methylation alteration before and after GC diagnosis. While, no significant difference was found probably due to the confounders difficult to control.

## Conclusions

In conclusion, our population-based nested case–control study found *COX-2* methylation in blood leukocyte DNA was at a low level, but may change during GC development. Further studies on methylation of specific genes in blood leukocyte DNA are needed for efficient biomarkers of GC early detection.

## Abbreviations

COX-2: Cyclooxygenase 2
CAG: Chronic atrophic gastritis
CI: Confidence interval
DYS: Dysplasia
GC: Gastric cancer
*H. pylori*: *Helicobacter pylori*
IM: Intestinal metaplasia
OR: Odds ratio
PCR: Polymerase chain reaction
QMSP: Quantitative methylation-specific PCR
SG: Superficial gastritis
